# Mycosynthesis of Silver Nanoparticles Using Screened *Trichoderma* Isolates and Their Antifungal Activity against *Sclerotinia sclerotiorum*

**DOI:** 10.3390/nano10101955

**Published:** 2020-09-30

**Authors:** Ali Athafah Tomah, Iman Sabah Abd Alamer, Bin Li, Jing-Ze Zhang

**Affiliations:** 1 Ministry of Agriculture, Key Lab of Molecular Biology of Crop Pathogens and Insects, Institute of Biotechnology, Zhejiang University, Hangzhou 310058, China; alialmalki775@yahoo.com (A.A.T.); emansabah29@yahoo.com (I.S.A.A.); Libin0571@zju.edu.cn (B.L.); 2 Plant Protection, College of Agriculture, University of Misan, AL-amarah 62001, Iraq; 3 Plant Protection, Agriculture Directorate, Maysan province, AL-amarah 62001, Iraq

**Keywords:** *Trichoderma virens*, gliotoxin, molecular interaction, antifungal activity, Sclerotia

## Abstract

To control the disease caused by *Sclerotinia sclerotiorum*, a total of 15 isolates of the *Trichoderma* species was screened for the biosynthesis of silver nanoparticles (AgNPs). Among them, the highest yield occurred in the synthesis of AgNPs using a cell-free aqueous filtrate of *T.virens* HZA14 producing gliotoxin. The synthetic AgNPs were charactered by SEM, EDS, TEM, XRD, and FTIR. Electron microscopy studies revealed that the size of AgNPs ranged from 5–50 nm and had spherical and oval shapes with smooth surfaces. Prepared AgNPs interacted with protein, carbohydrate and heterocyclic compound molecules, and especially, interaction patterns of AgNPs with the gliotoxin molecule were proposed. The antifungal activity assays demonstrated that percentage inhibition of the prepared AgNPs was 100, 93.8 and 100% against hyphal growth, sclerotial formation, and myceliogenic germination of sclerotia at a concentration of 200 μg/mL, respectively. The direct interaction between nanoparticles and fungal cells, including AgNPs’ contact, accumulation, lamellar fragment production and micropore or fissure formation on fungal cell walls, was revealed by SEM and EDS. These will extend our understanding of the mechanisms of AgNPs’ action for preventing diversified fungal disease.

## 1. Introduction

*Sclerotinia sclerotiorum* (Lib.) de Bary is a significant plant pathogenic fungus that leads to sclerotium disease on many crops and causes economical substantial losses throughout the world [1]. *S. sclerotiorum* has a broad host range including at least 408 described species of plant from 278 genera in 75 families [2]. The pathogen infects the host plants to cause diseases including cottony rot, watery soft rot, drop, crown rot, blossom blight, and perhaps most common, Sclerotinia stem rot (SSR) or white mold, and so on [1]. This pathogen produces the melanized multicellular structure, known as sclerotia that can survive for years in the soil [3]. The infection of host plants occurs from ascospores that originate from the germination of sclerotia in soil [1].

Diseases caused by *S. sclerotiorum* have traditionally been difficult to control due to a lack of high level resistance in major crops, which makes it difficult to improve resistance using classical breeding methods [4]. Disease management depends heavily on the application of fungicides, but this may cause environmental problems [5]. In addition, the emergence of *S. sclerotiorum* strains that are resistant to fungicides, such as carbendazim and benzimidazole in China, has been found [6].

Due to lack of more efficient disease control methods, this has stimulated researchers to develop new biotechnology. The nanoparticles (NPs) have been highly noticed due to the high bioactivity and broad antimicrobial spectrum with low doses [7]. While biogenic synthesis of NPs is considered to be an effective alternative for its non-toxic and green procedures [8], biosynthesis of NPs using organisms such as microorganism and plants, or their metabolisms has been explored and among them, silver nanoparticles (AgNPs) are favored for their broad-spectrum antimicrobial potential [9,10,11,12]. Especially, the use of fungi in biosynthesis of AgNPs is attractive due to the production of diversity proteins [13]. Among different fungi genera, biosynthesis of AgNPs using *Trichoderma* spp. is more convincing and safer for the environment in comparison with synthesis by other fungi [14,15,16,17,18,19,20]. Meanwhile, *Trichoderma* spp. produce rich metabolites, including different metabolic compounds, which depend on species or isolates, but less information was obtained regarding AgNPs and their interactions with proteins and metabolic compounds.

In addition, physiochemical properties of synthesized AgNPs differ in terms of their size, surface charge and shape, depending on different metabolites produced of different fungal species [13]. Although synthesized mechanisms have not yet been fully elucidated, interaction of AgNPs with fungal biomolecules is considered to be mainly responsible for the process of synthesis [13]. During this period, the toxic Ag^+^ ions are reduced to the non-toxic metallic AgNPs [16]. AgNPs synthesized by fungi have several effects on plant pathogens, including accumulation of Ag^0^ in transport systems, disturbance in the flow of ions and interruption of cellular processes (such as the respiration and metabolism pathways) [21]. However, the AgNPs synthesized by *T. harzianum* against the inhibition of mycelial growth and myceliogenic germination of sclerotia in *S. sclerotiorum* have been revealed but the mechanisms of its antifungal action were not evaluated [22].

Thus, the main purpose of this study was to screen *Trichoderma* isolates for efficient synthesis of AgNPs, to characterise AgNPs using scanning electron microscopy (SEM), energy dispersive spectroscopy (EDS), X-ray diffraction (XRD), transmission electron microscope (TEM) and fourier transform infrared spectroscopy (FTIR), and to assess the antifungal activity of biosynthesized AgNPs against plant pathogenic *S. sclerotiorum* and reveal its mode of action.

## 2. Materials and Methods

### 2.1. Fungal Isolates and Mycelial Growth

Fifteen Trichoderma isolates, including the *T. atroviride* HZA1, *T. atroviride* HZA2, *T. afroharzianum* HZA3, *T. brevicompactum* HZA4, *T. dorothopsis* HZA5, *T. koningiopsis* HZA6, *T. brevicompactum* HZA7, *T. dorothopsis* HZA8, *T. citrinoviride* HZA9, *T. asperellum* HZA10, *T. harzianum* HZA11, *T. brevicompactum* HZA12, *T. atroviride* HZA13, *T. virens* HZA14, and *T. dorothopsis* HZA15, were used in this study. They were isolated and identified from previous studies in this lab [23]. Plant pathogenic fungus Sclerotinia sclerotiorum YY01 was a highly aggressive isolate and it was previously isolated from the diseased tuber mustard (Brassica juncea var. tumida), identified and deposited in the Culture Collection of the Biotechnology Institute, Zhejiang University, Zhejiang Province, China.

Fifteen *Trichoderma* isolates were grown in 250 mL Erlenmeyer flasks containing 100 mL liquid broth consisting of (g/L) KH_2_PO_4_, 7; K_2_HPO_4_, 2; MgSO_4_ × 7H_2_O, 0.1; (NH_2_)SO_4_, 1; yeast extract, 0.6; glucose, 10 [24], which were incubated in a ZWY-211B rotating shaker at 25 °C and 150 rpm for 72 h. Subsequently, the mycelia were collected after filtrating them with the clean cloth–cheese, and washed using sterile double-distilled water (ddH_2_O), before they were used for the synthesis of silver nanoparticles (AgNPs).

### 2.2. Synthesis of Silver Nanoparticles Using Trichoderma Isolates.

For the biosynthesis of AgNPs, 10 g of mycelia (fresh weight) for each of the *Trichoderma* isolates were put into a 250 mL flask containing 100 mL of sterile ddH_2_O and incubated in a rotating shaker at 25 °C and 150 rpm for 96 h [25]. The cell-free filtrate was obtained for each isolate after moving mycelia with Whatman filter paper No.1 and adjusting the pH to 7.0. The filtrate for each isolate was mixed with 1mM of AgNO_3_ solution, which was prepared by Silver nitrate (AgNO_3_) from Sinopharm Chemical Reagent Co., Ltd. (Shanghai, China), and dissolved in distilled water as a silver source for AgNPs biosynthesis [19]. Reaction mixtures were incubated in a rotating shaker at 25 °C and 150 rpm in dark conditions for 1 h [15]. All experiments were done in triplicate. The color change of each reaction liquid was observed, and the isolates were selected based on the reaction liquid with a strong color change for re-synthesizing the AgNPs. The synthesis procedure and conditions were the same as described above but the time of synthesis was kept for 24, 48, 72, 96 and 120 h, respectively. The experimental flasks without the silver ion but with mycelia were used for control and the experiments were done in triplicate. To ensure the formation of AgNPs, the absorbance spectra (UV–Visible) of colloidal suspension were measured as a wavelength function using a UV2550 UV-Vis spectrophotometer (Shimadzu, Kyoto, Japan) at 1.0 nm of data intervals. The obtained colloidal suspension of AgNPs was recovered by centrifuging (20,000 rpm) it for 10 min and repetitively washing with distilled ddH_2_O to remove the silver ion residue. These precipitates were freeze-dried by Alpha 1-2 LDplus dryer (Osterode, Germany) into AgNPs powder for characterization study.

### 2.3. Characterization of AgNPs

The surface morphology and size of the produced AgNPs were observed using a Su8010 scanning electron microscope (SEM) (HITACHI, Japan). The energy dispersive spectroscopy (EDS) of Nano-silver elements’ density was confirmed using an Oxford instruments EDS (X-Max^N^ 80, Oxford, UK) at 20 keV in conjunction with scanning electron microscopy (SEM). The shape and size of AgNPs were detected by using a JEM-1010 transmission electron microscope (TEM) (JEOL USA Inc., Peabody, MA, USA). To prepare the AgNPs sample for TEM, copper grid was dipped in AgNPs solution and dried under vacuum. The crystalline nature of AgNPs powder was detected by X-ray Diffraction (XRD) analysis using a Siemens D5000 diffractometer (Munich, Germany) with an operating voltage of 45 kV and a current of 0.8 mA.

The FT-IR spectra were recorded to identify the possible biomolecules responsible for the reduction of the Ag^+^ ions and capping of the bioreduced AgNPs synthesized by the *T. virens* extract. The interaction between functional groups of cell-free extract with AgNPs was analyzed using a Fourier transform infrared (FTIR) spectrometer (Bruker Vector 22, Ettlingen, Germany), where dried powder of fungal extract and AgNPs were mixed with KBr crystals, respectively, and compressed to form pieces for analysis in the mid-infrared light region of 490–4000 cm^−1^ with a resolution of 4 cm^−1^. In addition, purified gliotoxin powder, obtained from *T. virens* HZA14 [23], was also analyzed by FTIR spectra as control.

### 2.4. Inhibitory Activity of Synthetic AgNPs against Mycelial Growth

To investigate the effect of AgNPs synthesized by *T.virens* HZA14 against mycelial growth of *S. sclerotiorum*, the AgNPs’ mother solution (1000.0 μg/mL) was prepared after being dissolved in sterile ddH_2_O by using ultrasonic waves [26]. The series solution of AgNPs’ solutions were mixed with the melted PDA after the mother solution was diluted, and the plates containing the AgNPs of different concentrations (50, 100, 150, and 200 μg/mL) were obtained. A disc (5 mm) containing hyphae of *S. sclerotiorum* from 5-day-old colonies was put into the center of a plate, which was incubated at 23 °C for 10 days. The 1.0 mL fungal cell-free culture liquid (CFCL) diluted by twice as much, or sterile, ddH_2_O was mixed with the melted PDA, as with the controls. All experiments were done in triplicate. Diameters of colonies were measured and the percentage inhibition (PI) was calculated using the formula PI (%) = [(C − N)/C)] × 100, where I was the percentage inhibition of the radial growth of *S. sclerotiorum* hyphae, C was the colony diameter in the plate with sterile ddH_2_O and N was the colony diameter in the plate with AgNPs or 50% CFCL.

### 2.5. Effect of AgNPs on Sclerotial Production

For testing the effect of biosynthetic AgNPs on the sclerotial production of *S. sclerotiorum*, plates containing PDA were covered with the sterile cellophane membrane. A disc (3 mm) containing the 5-day-old hyphae of *S. sclerotiorum* was put into the center of a plate and incubated at 23 °C for 4 days and the cellophane membranes with colonies were transferred into the plates containing PDA with AgNPs of different concentrations (50, 100, 150, and 200 μg/mL), 50% CFCL and sterile ddH_2_O, as described above. The plates were incubated at 23 °C for 15 days. The number of sclerotia was counted on each plate and the percentage inhibition of produce using the same formula that was described above.

### 2.6. Determination of Effect of AgNPs on, Myceliogenic Germination of Sclerotia

Antifungal effects of the synthetic AgNPs on sclerotial viability were also tested in vitro using the sterile soil [27]. The 20 g of sterilized soil (peat: farmyard soil in a 2:1 ratio) was put in sterile glass plates (9 cm in diameter). Sterilized sclerotia were moved randomly into the soil surface, and then the plates were sprayed using 20 mL AgNPs solutions of different concentrations (50, 100, 150, and 200 μg/mL) or 50% CFCL or sterile ddH_2_O. Plates were sealed by parafilm strips and incubated at 23 °C in dark conditions for five days. Sclerotia were surface-sterilized in 50% ethanol for two minutes, and dried on sterile filter paper under a laminar flow hood after rinsing with sterile water. Partial sclerotia were placed into the plate containing PDA and incubated at 23 °C for three days. Sclerotia producing hyphae were counted and the percentage of germination inhibition was evaluated using the same formula as described above.

To observe the interaction of AgNPs with fungal hyphae cells, sclerotia incubated for five and seven days were fixed in glutaraldehyde solution of 2.5% overnight for SEM observation, as described by [28]. Briefly, the samples were washed prior to post fixation in 1% (w/v) osmium tetroxide and dehydrated in a graded ethanol series (50%, 70%, 80%, 90%, 95%, and 100%). Before the critical-point drying, the samples were mounted onto an aluminum stub, sputter-coated with gold and examined and photographed in a SEM (SU8010, HITACHI, Japan). The Nano-silver elements’ density was confirmed using the Oxford Instruments energy dispersive spectroscope (EDS) (X-Max^N^ 80, Oxford, UK) at 20 keV, as described above.

## 3. Results

### 3.1. Synthesis of Nanoparticles 

**Figure 1:**
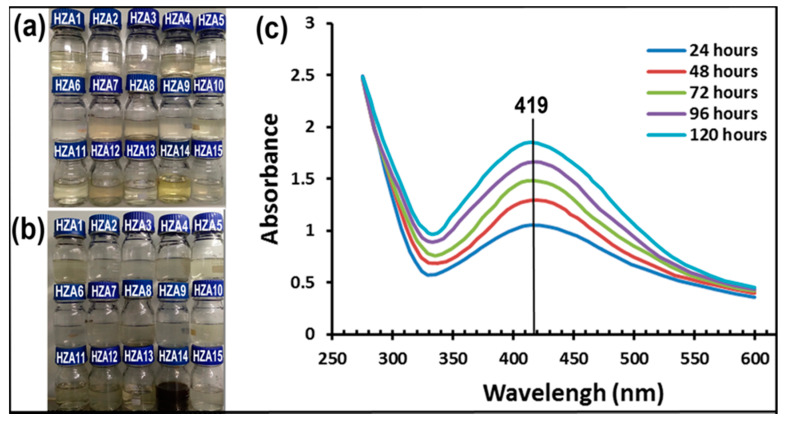
Biosynthesis of AgNPs using 15 *Trichoderma* spp. isolates and UV-Visible spectra of synthetic AgNPs using cell-free filtrate of *T. virens* HZA14: (**a**) Cell-free filtrate produced by *Trichoderma* spp. isolates in different bottles; (**b**) Synthetic AgNPs suspensions in different bottles after 120 h of incubation. The dark brown suspension in a bottle shows the formation of AgNPs; (**c**) UV-Visible spectra of synthetic AgNPs after different times of incubation.

The primary investigation of AgNPs formation was confirmed using visual color change and UV-visible spectroscopy. The experiment results showed that the color change markedly occurred from pale yellow to dark brown only in the reaction mixture produced by *T. virens* HZA14 among 15 isolates (Figure 1a,b), indicating the formation of maximum AgNPs after 120 h of incubation. Subsequently, the screened isolate HZA14 was used for the synthesis of AgNPs after different incubation times (24, 48, 72, 96 and 120 h). Analysis of the UV-Visible spectra revealed that the sharp surface plasma resonance peaks were all observed at 419 nm after different incubation times (Figure 1c). Obviously, peaks were the representative characteristic of AgNPs formation. Meanwhile, with time extension of incubation, enhancement of absorption increased and the maximum absorbance peak was observed after 120 h (Figure 1c). There was no obvious color alteration and absorbance peak observation at 419 nm in the control solution with cell-free filtrate but without AgNO_3_ after 120 h of incubation.

### 3.2. Characterization of AgNPs

**Figure 2:**
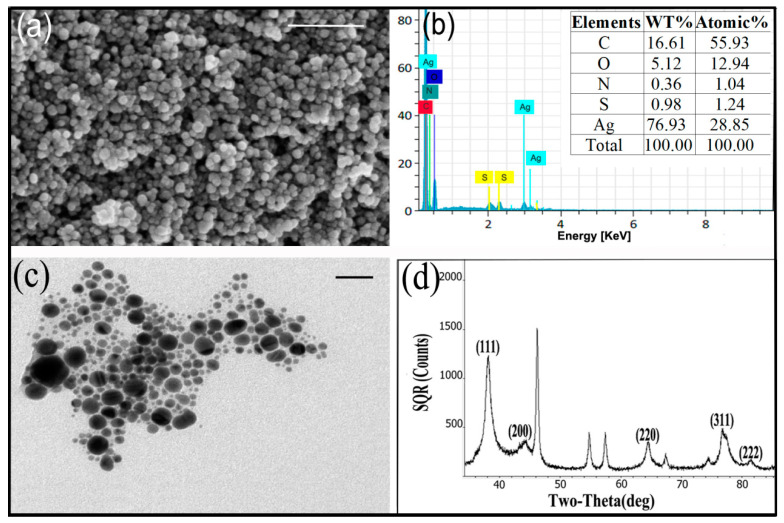
Characterization of the synthesized AgNPs after 120 h of incubation: (**a**) Scanning electron microscopy. Scale bar = 250 nm; (**b**) Energy dispersive spectroscopy; (**c**) Transmission electron microscopy. Scale bar = 50 nm; (**d**) X-ray diffractogram pattern.

The prepared AgNPs were characterized by scanning electron microscopy (SEM), energy dispersive spectroscopy (EDS), transmission electron microscopy (TEM), X-ray diffraction (XRD) and Fourier transform infrared (FT-IR) spectroscopy analysis. The SEM micrographs showed that the external surfaces of spherical AgNPs synthesized by cell-free filtrate of *T. virens* HZA14 were smooth (Figure 2a). The EDS analysis of AgNPs revealed the pure silver (28.85%) at 3 KeV was the second major constituent element compared to oxygen, nitrogen, carbon, and sulphur elements (Figure 2b), confirming the existence of the silver element in the synthesized AgNPs. In addition, the EDS data also indicated that the relative proportion (1.24%) of the sulphur element was higher than that (1.04%) of the nitrogen element, which was an unusual finding. The TEM micrographs showed that the different size and shape of AgNPs ranged from 5 to 50 nm (Figure 2c). The AgNPs exhibited a difference in their size but the majority of nanoparticles were spherical, while others were oval-shaped. Occasionally, irregular and larger sized nanoparticles are also visualized in Figure 2c. Furthermore, XRD analysis demonstrated that the emission peaks at 2*θ* values of 38.2, 44.2, 64.6, 77.5 and 81.5 corresponded to the silver crystal planes (111), (200), (220), (311) and (222), respectively (Figure 2d), confirming the crystalline nature of AgNPs. The XRD pattern thus clearly illustrates that the synthesized AgNPs were crystalline in nature as well as monodispersed in colloidal form. The line broadening of peaks was related to the presence of small particles in the medium. A few unassigned peaks were also observed in the vicinity of the characteristic peaks (Figure 2d). The unassigned peaks were recorded in the XRD pattern and they could be due to the crystallization of the bioorganic phase that occurs on the surface of the nanoparticles [29].

**Figure 3:**
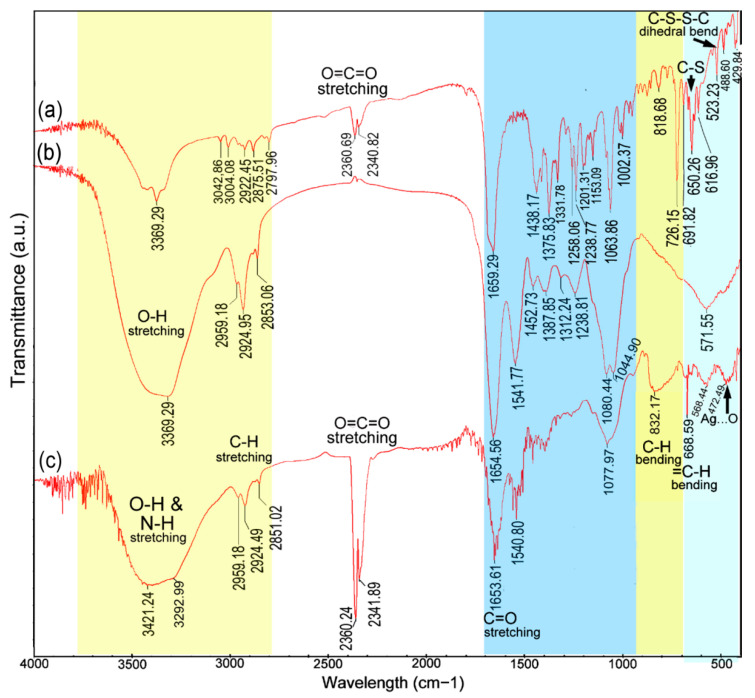
FT-IR spectra of three samples: (**a**) Gliotoxin; (**b**) *T. virens* extract; (**c**) AgNPs.

FTIR measurements were carried out for identifying the functional groups involved in the AgNPs’ reduction (Figure 3). The broad and strong bands at 3421 cm^−1^ and 3292 cm^−1^ were due to the bonded amine groups (–NH) or hydroxyl (–OH) in the interaction of fungal extract with AgNPs powder, respectively [30]. The peaks that appeared at 2959, 2923 and 2851 cm^−1^ were attributed to asymmetric CH_3_, and symmetric and asymmetric CH_2_, stretching modes of carbohydrates and fatty acids. The peaks at 2360 and 2340 cm^−1^ were due to O=C=O stretching vibrations (CO_2_). The peaks at 1653, 1540 and 1077 cm^−1^ were attributed to the C=O (carboxylic group), C=C–C (aromatic ring or amide II group) and C–O or C–O–C stretching vibrations, respectively [31]. A stronger absorption peak at 832 cm^−1^ was assigned to the C–H bending vibration that was adjacent to the substituent group, indicative of heterocyclic compounds secreted by *T. virens*, which acted as the capping agent [32]. The peaks for the binding of the C–S–H groups were assigned at 668 cm^−1^. The peaks for the O–C and P–O–C groups in phospholipids, aromatics, amino acids (rocking vibrations) and ketones could be assigned near 568 cm^−1^ [8]. Similarly, the absorption band at near 472 cm^−1^ was attributed to the AgNPs binding with oxygen [31,33].

Characteristics of FTIR spectra and the shift changes in AgNPs powder are presented in Table 1. The peak at 3292 cm^−1^ with a large shift change (−76.20 cm^−1^, compared to fungal extract) revealed the AgNPs binding strongly with oxygen from the oxidized form of gliotoxin or negatively charged carboxyl groups in proteins. Secondly, the peak of the C–S–H group’s stretching vibrations, with remarkable shift change (+18.33 cm^−1^, compared to the FT-IR spectrum of gliotoxin), was assigned at 668 cm^−1^ and suggested that the AgNPs were binding with sulphur from the reduced forms of gliotoxin [34]. This could be confirmed by obvious shift change (+13.49 cm^−1^) of the peak at 832 cm^−1^ for the C–H bending vibration being adjacent to the substituent group. In addition, the peak of the R–CO–NH_2_ stretching vibrations near 1077 cm^−1^ also revealed the presence of proteins in synthetic AgNPs [32]. These biological components may play a significant role in the formation and stabilization of nanoparticles [8].

### 3.3. Inhibitory Activity against Hyphal Growth

**Figure 4:**
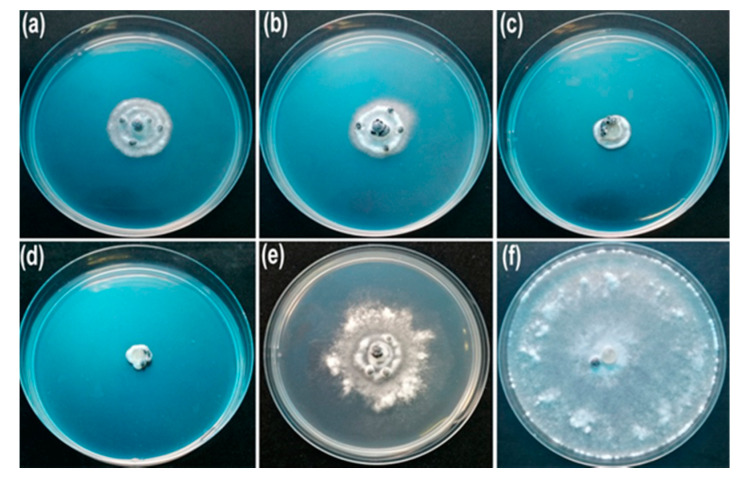
Effect of AgNPs on the hyphal growth of *S. sclerotium* on PDA 10 days after inoculation: (**a**) 50 μg/mL; (**b**) 100 μg/mL; (**c**) 150 μg/mL; (**d**) 200 μg/mL; (**e**) 50% cell-free culture liquid (CFCL) of *T. virens* HZA14; (**f**) ddH_2_O.

The inhibitory activity of AgNPs synthesized by the cell-free filtrate produced by *T. virens* HZA14 against hyphal growth of *S. sclerotiorum* was assessed. The results showed that the AgNPs significantly inhibited the growth of fungal hyphae (Figure 4). The highest percentage inhibition (PI %) of colony diameters was 100% at a concentration of 200 μg/mL, followed by 82.75, 72.03 and 66.70% at the concentrations of 150, 100, and 50 μg/mL (*p* < 0.05), respectively (Table 2). In addition, 50% cell-free culture liquid (CFCL) of *T. virens* HZA14 also had markedly antifungal activity compared with control (ddH_2_O).

### 3.4. Inhibitory Activity against Sclerotial Production

**Figure 5:**
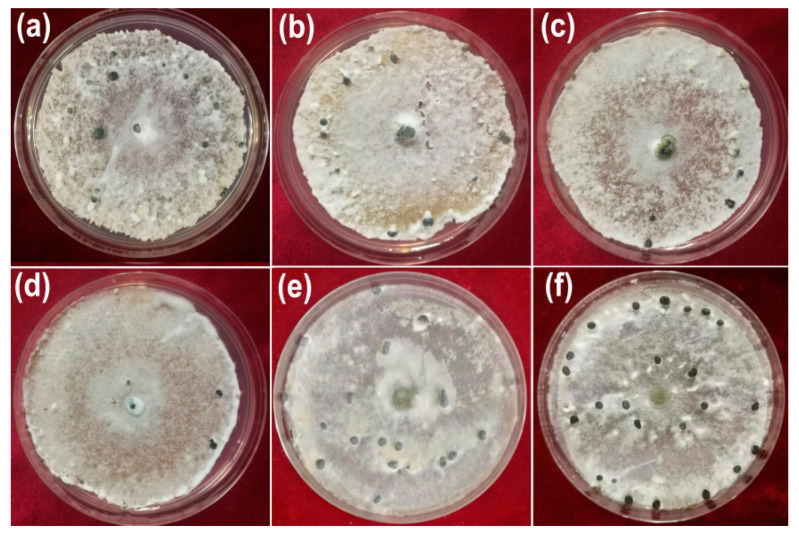
Effect of AgNPs on the sclerotia production of *S. sclerotium* 15 days after inoculation: (**a**) 50 μg/mL; (**b**) 100 μg/mL; (**c**) 150 μg/mL; (**d**) 200 μg/mL; (**e**) 50% cell-free culture liquid (CFCL); (**f**) ddH_2_O.

To determine inhibitory activity of AgNPs against sclerotial production, cellophane membranes (nine cm in dimeter) containing hyphae of *S. sclerotiorum* were covered onto the plates containing PDA with the AgNPs of different concentrations (50, 100, 150, and 200 μg/mL) for 15 days. The results showed that the synthesized AgNPs significantly reduced the number of sclerotia produced (Figure 5). The percentage inhibition (PI) of sclerotia production was 93.81, 76.33, 54.63 and 23.7% at concentrations of 200, 150, 100 and 50 μg/mL, respectively (Table 2). By comparison, the PI of 50% cell-free culture liquid (CFCL) was 18.55% compared with control (ddH_2_O).

### 3.5. Inhibitory Activity against Myceliogenic Germination of Sclerotia

**Figure 6:**
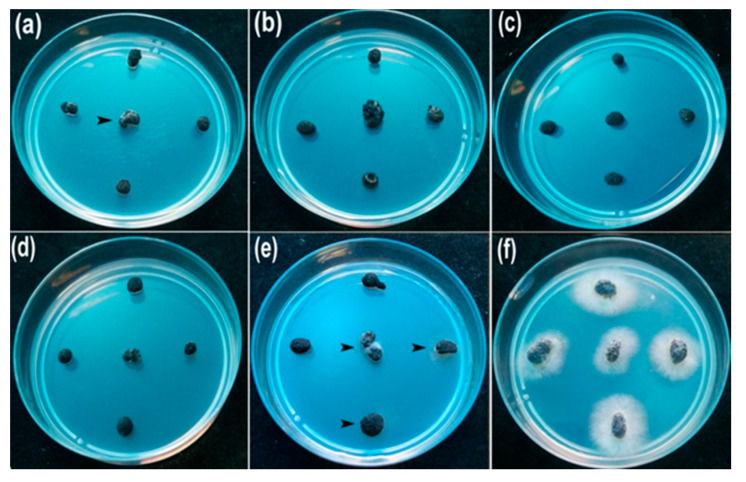
Effect of different concentrations of AgNPs on myceliogenic germination of sclerotia on PDA for 3 days: (**a**) 50 μg/mL; (**b**) 100 μg/mL; (**c**) 150 μg/mL; (**d**) 200 μg/mL; (**e**) 50% cell-free culture liquid (CFCL); (**f**) ddH_2_O.

For determining the inhibitory activity of AgNPs against myceliogenic germination of sclerotia (MGS), 20 mL AgNPs suspension with different concentrations (50, 100, 150, and 200 μg/mL) were sprayed on sclerotia on the surface of the soil and treated at 23 °C for five days. The results of myceliogenic germination of sclerotia on PDA indicated that the MGS was completely inhibited after treatment with three different concentrations of AgNPs (100, 150 and 200 μg/mL AgNPs) (Figure 6). The 80% PI of the MGS was observed at a concentration of 50 μg/mL AgNPs (*p* < 0.05) (Table 2). Comparatively, 50% CFCL also showed the higher inhibitory activity against the MGS (PI of 45%).

**Figure 7:**
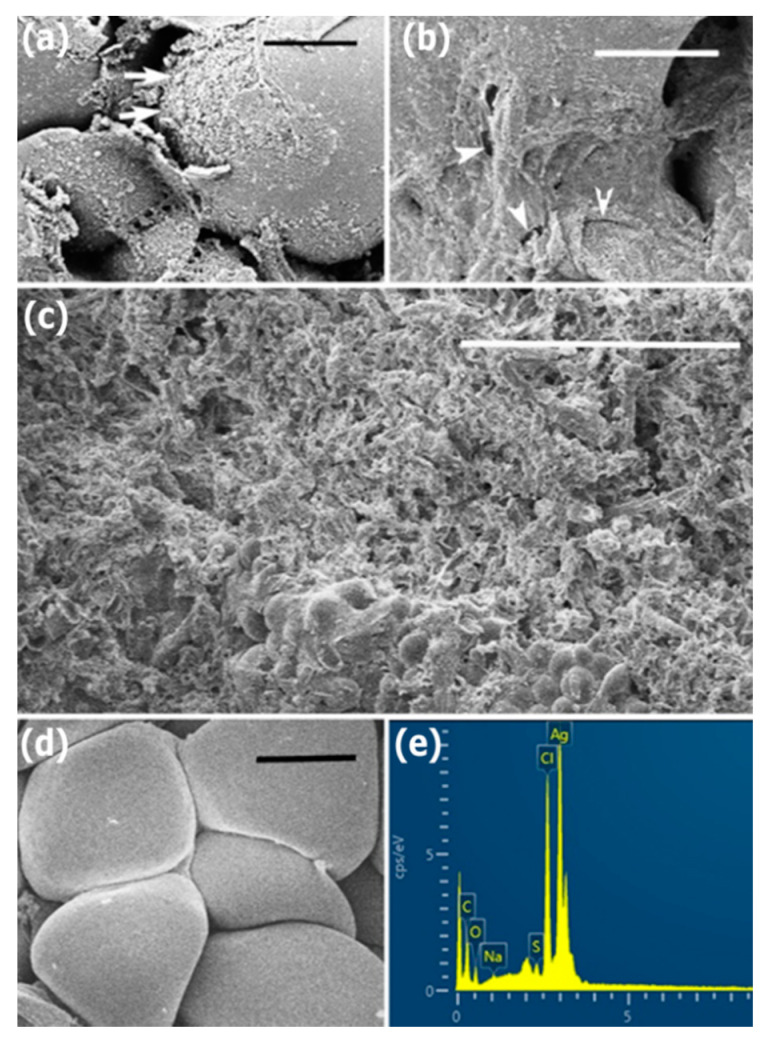
The micrographs of scanning electron microscopy and energy dispersive spectroscopy, after sclerotia were treated by 200 μg/mL AgNPs: (**a**,**b**) Five days; (**a**) Lamellar fragments (arrows) on the surfaces of hyphae cells. Scale bar = 4 μm; (**b**) Micropores or fissures (arrowheads) on the surfaces of hyphae cells. Scale bar = 4 μm; (**c**) Seven days. Mycelia die on the surfaces of a sclerotium. Scale bar = 100 μm; (**d**) Control. Hyphae cells indicate regular shapes on the surfaces of a sclerotium. Scale bar = 5 μm. (**e**) Energy dispersive spectroscopy.

To observe the interaction of the AgNPs with hyphae cells, morphological changes on the surfaces of sclerotia were seen by SEM after sclerotia were treated at a concentration of 200 μg/mL AgNPs for five or seven days. The micrographs of SEM showed that the small lamellar fragments (Figure 7a) or micropores or fissures (Figure 7b) appeared on the surfaces of hyphae cells on samples treated for five days, revealed damage of hyphae cells’ integrity. After seven days, complete collapse of hyphae cells occurred on the surface of a sclerotium (Figure 7c). By comparison, the regular hyphae cell structure was observed on the control samples (Figure 7d). The energy dispersive spectroscopy (EDS) analysis indicated the presence and accumulation of Ag as well as O, N, C, and S elements on hyphae cell surfaces of the sclerotia on samples treated for five days (Figure 7e).

## 4. Discussion

AgNPs have unique optical, electrical, and thermal properties, which constitute the basis of novel applications in antimicrobial, anticancer, larvicidal, catalytic, and wound healing activities [35]. They are able to adhere to the cell walls and membranes of microorganisms and then may gain access to the cell interior, leading to cellular structural damage, production of reactive oxygen species, and interruption of the signaling pathway [36,37]. These properties enable AgNPs to become promising application prospects in the control of pathogenic microorganisms in the areas of health and agriculture [38].

With research and development in the synthesis of nanoparticles, these activities have attracted increasing interest in biogenic synthesis methods using organisms such as bacteria, fungi, and plants, or the byproducts of their metabolism due to lower toxicity, better physicochemical characteristics, and higher stability [39]. However, although various organisms have potential for use in biogenic synthesis, the synthetic yield has a considerable difference, depending on the species or strain of the organism [38]. In this study, the highest AgNPs formation occurred in the synthesis of AgNPs using the *T. virens* HZA14, showing great difference among isolates of different species (Figure 1).

**Figure 8:**
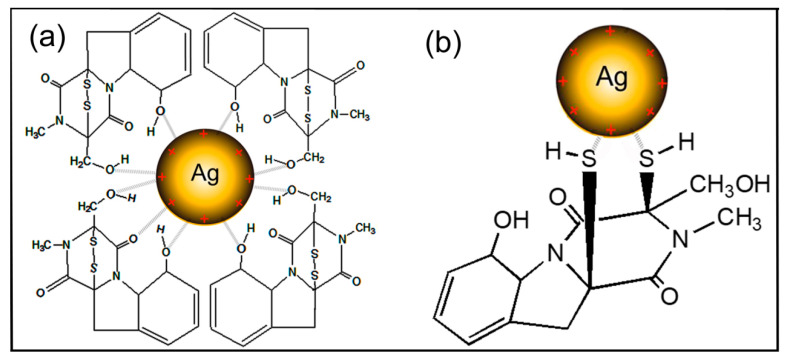
Schematic illustration showing the interactions between the oxidized (**a**) or reduced (**b**) form of gliotoxin with the surface of positive charge of silver nanoparticles (AgNPs).

The synthetic yield was involved in components of secondary metabolites secreted by *Trichoderma* spp. and synthesis mechanisms of AgNPs. The isolate HZA14 produces gliotoxin, which has been identified previously [23]. Characterizations of AgNPs synthesized using the *T. virens* HZA14 revealed that the gliotoxin took part in the synthesis of AgNPs. The EDS data also indicated that the relative proportion (1.24%) of the sulphur element was higher than that (1.04%) of the nitrogen element. FTIR study revealed the presence and binding of protein, carbohydrates and heterocyclic compounds and fatty acids with AgNPs. It is well known that the proteins bind with nanoparticles through cysteine residues or free amino groups [40]. Electrostatic interactions of negatively charged carboxyl groups in proteins or in heterocyclic compounds also bind with nanoparticles [8]. However, the peak with a larger shift change revealed the AgNPs binding strongly with oxygen from oxidized or reduced forms of gliotoxin (Table 1). Therefore, interaction patterns for the AgNPs binding negatively charged carboxyl groups or dithiol groups in gliotoxin are proposed and the schematic illustration of the synthesis of AgNPs capped with gliotoxin is depicted in Figure 8. Although a previous study also found that the largest amount of AgNPs could be biosynthesized by *T. virens* [41], the interaction of AgNPs with fungal metabolites was not characterized. In the biosynthesis of AgNPs using *T. longibrachiatum* [20], synthesized AgNPs interacted only with proteins by carbonyl groups of amino acid residues and peptides, while, in this study, interaction patterns of AgNPs with metabolites, especially with gliotoxin, provide new insight for understanding the synthesized mechanisms of AgNPs.

The AgNPs exhibit their antimicrobial potential through multifaceted mechanisms [37]. It is interesting to note that AgNPs are predominantly used for plant disease management due to their antimicrobial activity against a diverse and broad range of plant pathogens [42]. In this study, synthesized AgNPs exhibited high inhibitory activity against hyphal growth, sclerotial formation, and myceliogenic germination of sclerotia of *S. sclerotium*, showing their potential applications against white mold, which is partially similar to a previous report [22]. However, in this study, the mode of AgNPs on fungal hyphae was characterized by SEM based on the morphological changes of fungal hyphae on the sclerotia and the presence of nanoparticles was confirmed by EDS, providing evidence of direct physical interaction between nanoparticles and fungal cells. These findings revealed that direct physical interaction of AgNPs with fungal hyphal cells causes an alteration in the fungal cell wall structure, including AgNPs contact, accumulation, lamellar fragments and micropore or fissure formation, possibly allowing AgNPs into the cell interior. Similar works also found that the antifungal role of nanoparticles arose from an initial direct contact with fungal cell walls, inducing ROS production, destroying membrane integrity, and altering morphological characteristics [37,43]. However, the gliotoxin is an epipolythiodioxopiperazine class toxin, containing a disulfide bridge, with high agonistic activity against plant pathogens [23]. Whether or not the prepared AgNPs with gliotoxin possess higher inhibitory activity than those without gliotoxin needs to be confirmed further. Therefore, this study will extend our understanding of nanoparticles that could potentially be adopted as an effective strategy for preventing diversified fungal disease. The use of AgNPs in the form of nanopesticides in agroecosystems is not fully explored, and further research should focus on its risk assessment, such as using Quantitative Structure–Activity Relationship/Quantitative Structure–Property Relationship (QSAR/QSPR) tools [44], for achieving safer and more efficient agricultural practices.

## 5. Conclusions

In conclusion, this study demonstrated for the first time the prominent antifungal activity of AgNPs synthesized using a cell-free aqueous filtrate of *T. virens* HZA14 producing gliotoxin against the soilborne pathogen *S. sclerotiorum* in vitro. Characterization of AgNPs revealed that the highest yield was related to gliotoxin produced by *T. virens* HZA14, and meanwhile, interaction patterns of AgNPs with gliotoxin molecules were proposed. The biosynthesized AgNPs showed a high percentage inhibition against hyphal growth, sclerotial formation, and myceliogenic germination of sclerotia. All the inhibitory behaviors observed using SEM/EDS technologies may be mostly attributed to nanoparticle–cell direct contact, accumulation, lamellar fragment production and micropore or fissure formation on fungal cell walls and may be related to the alteration of fungal cell walls, while these changes provide the channels for the AgNPs to enter the cells. The final cell death may be associated with cell membrane damage, oxidative stress, and altered transport activity and signal transduction pathways.

## Figures and Tables

**Table 1:** Fourier-transform infrared spectral characteristics of fungal extract and AgNPs powder.

No.	Description	Wavenumber (cm^−1^)		Differences	Possible Reason for the Shift Alteration
		Fungal Extract	AgNPs Powder		
**1**	N–H stretching vibrations	–	3421.24	–	Interaction with proteins
**2**	O–H stretching vibrations	3369	3292.99	−76.20	Interaction with proteins or negatively charged carboxyl groups in gliotoxin
**3**	C–H stretching vibration (alkanes)	2853.06	2851.02	−2.04	Interaction with fatty acids and carbohydrates
**4**	CO_2_ stretching vibrations	–	2360.24 and 2341.89	–	An increase in carbon dioxide in the extract or poor purge stability of the instrument
**5**	Carbonyl group (C=O), amide I group stretching vibrations	1654.56	1653.61	−0.95	Binding with proteins
**6**	Amide II group stretching vibrations	1541.77	1540.80	−0.97	Interaction with proteins
**7**	P=O stretching in phospholipids and the C=O group in polysaccharides	1080.44	1077.96	−2.48	Interactions with lipids and carbohydrates
**8**	C–H bending vibration that was adjacent to the substituent group	–	832.17	+13.49 ^1^	Interactions with heterocyclic compounds
**9**	C–S–H group stretching vibrations	–	668.59	+18.33 ^1^	Ag binding with sulfur from the reduced forms of gliotoxin
**10**	C–O–C and P–O–C on phospholipids, aromatics, amino acids and ketones stretching vibrations	571.55	568.44	−3.11	Interactions with phospholipids, aromatics, amino acids and ketones.
**11**	O–Ag stretching vibrations	–	472.49	–	Ag binding with oxygen from hydroxyl groups of gliotoxin

^1^ comparing with gliotoxin.

**Table 2:** Effect of AgNPs of different concentrations against the hyphal growth (HG), sclerotia production (SP), and myceliogenic germination of sclerotia (MSG) of *S. sclerotiorum.*

Treatments	HG (mm)	PI (%) ^1^	SP (no.)	PI% ^2^	MSG (no.)	PI% ^3^
**AgNPs (50 μg/mL)**	28.31 ± 0.8	66.70 ± 0.9 d	18.50 ± 0.5	23.70 ± 2.3 d	1.00 ± 0.8	80 ± 16.0 b
**AgNPs (100 μg/mL)**	23.78 ± 1.1	72.03 ± 1.3 c	11.00 ± 0.8	54.63 ± 3.3 c	0.00 ± 0.0	100 ± 0.0 a
**AgNPs (150 μg/mL)**	14.66 ± 0.1	82.75 ± 0.1 b	5.75 ± 0.9	76.33 ± 3.4 b	0.00 ± 0.0	100 ± 0.0 a
**AgNPs (200 μg/mL)**	0.0 ± 0.0	100 ± 0.0 a	1.50 ± 0.5	93.81 ± 2.0 a	0.00 ± 0.0	100 ± 0.0 a
**Tv-CFA (50%)**	64.75 ± 0.4	23.82 ± 0.4 e	19.75 ± 0.9	18.55 ± 3.8 e	2.75 ± 0.5	45 ± 9.7 c
**ddH_2_O**	85.00 ± 0.0	-	24.25 ± 0.9	-	5.00 ± 0.0	-

^1^ PI: percentage inhibition of colony diameter; ^2^ PI: percentage inhibition of sclerotia production; ^3^ PI: percentage inhibition of sclerotia germination. The different letters are significantly different (*p* < 0.05) by LSD test; values are the average of four replicates.
